# Supplementary material for: Elevation shapes alpine snow algal blooms and their influence on albedo reduction
Source: New Phytol. 2026 Jun 25;251(5):2482–96. doi: 10.1111/nph.71360 (PMC13443302; doi:10.1111/nph.71360)

New Phytologist Supporting Information

Article title: Elevation shapes alpine snow algal blooms and their influence on albedo reduction

Authors: Pablo Almela, Scott Hotaling, J. Joseph Giersch, Helena C. L. Klip, James J. Elser, Trinity L. Hamilton

Article acceptance date: 26 May 2026

Supplementary Figure S1. Spatial distribution of sampling sites across the study regions. Points represent individual samples and are colored by (A) elevation (m a.s.l), (B) day of year (DOY), and (C) snow depth (cm).

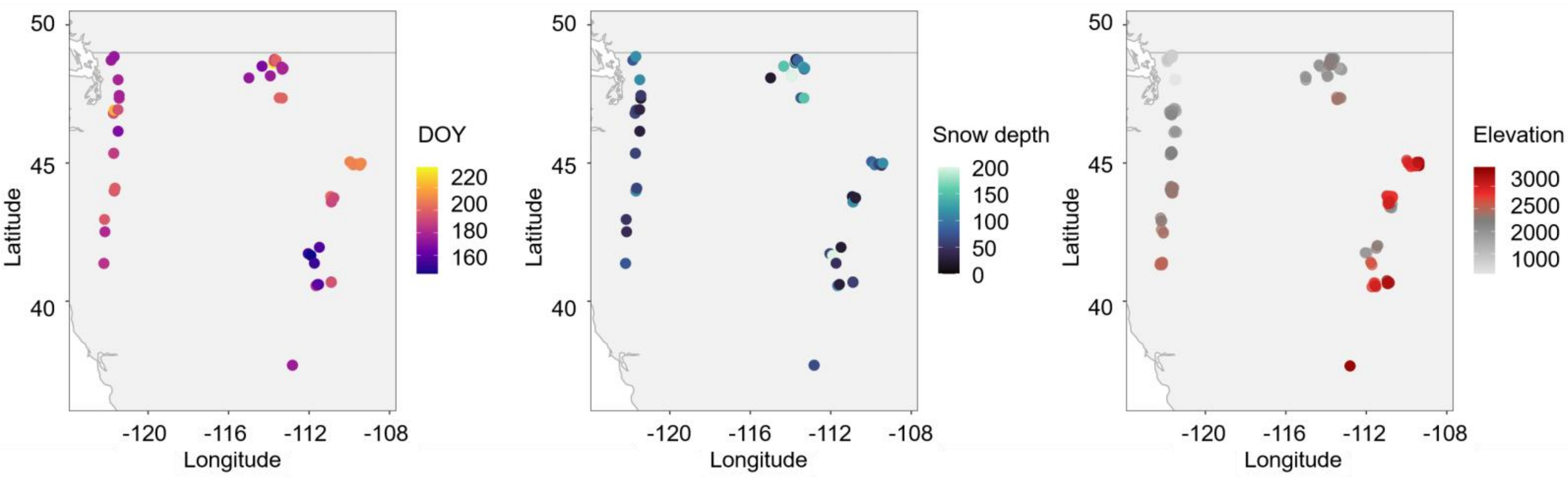

Supplement: Supplementary file 1 [file NPH-251-2482-s001.pdf]
